# Profile of forensic patients hospitalized in psychiatry

**DOI:** 10.1192/j.eurpsy.2023.1134

**Published:** 2023-07-19

**Authors:** F. Azraf, H. Berrada, S. Belbachir, A. Ouanass

**Affiliations:** Arrazi Hospital, Faculty of Medicine and Pharmacy of Rabat, sale, Morocco

## Abstract

**Introduction:**

The question of violence and dangerousness is a central subject in psychiatry, since it is a source of stigmatization for many patients and represents a major element of medical, legal and social care. Violence being defined as: "the deliberate use or threat of deliberate use of physical force or power against oneself, against another person or against a group or community, which results in or is very likely to lead to trauma, death, moral damage, maldevelopment or deficiency”. The violent character of the mentally ill was among the precursors to the discussion around the first therapeutic measures for the mentally ill. A better knowledge of the risk factors for the passage to a violent act in mental disorders is therefore necessary for the development of therapeutic and preventive strategies.

**Objectives:**

The objective of our work is to determine the socio-demographic, clinical and criminological characteristics of forensic patients and to seek the predictive factors of violence in these patients.

**Methods:**

This is a retrospective study spread over 10 years on a series of forensic patients who were hospitalized at the Ar-razi psychiatric hospital in Salé (Morocco). The collection of data was carried out from clinical observations, psychiatric expertise and an exploitation sheet.

**Results:**

Numerous studies have defined subgroups of patients at risk. Factors such as positive symptoms, poor treatment compliance, and comorbidities such as substance use disorders or antisocial personality traits have been described as predictive of violence.

**Conclusions:**

The prevention of medico-legal acts must take into consideration these associated factors, in particular early management of the disorders, improvement of therapeutic observance and family care.

**Disclosure of Interest:**

None Declared

